# Toward Robust Machine Learning Models for MALDI-TOF MS: Novel Approaches for *Mycobacterium abscessus* Subspecies Identification

**DOI:** 10.1021/acs.jproteome.5c00534

**Published:** 2026-02-09

**Authors:** Erica Padial-Fuillerat, Juan E. Martínez-Manjón, Igor Zwir, Manuel J. Arroyo, Mario Blázquez-Sánchez, David Rodríguez-Temporal, Belén Rodríguez, Luis Mancera, Coral del Val

**Affiliations:** † Clover Bioanalytical Software S.L, Granada 18016, Spain; ‡ Department of Computer Science and Artificial Intelligence, Andalusian Research Institute in Data Science and Computational Intelligence (DaSCI), 16741 University of Granada, Granada 18071, Spain; § Instituto de Investigación Biosanitaria ibs.GRANADA, Complejo Hospitales Universitarios de Granada, Universidad de Granada, Granada 18012, Spain; ∥ Clinical Microbiology and Infectious Diseases Department, 16483 Hospital General Universitario Gregorio Marañón, Madrid 28007, Spain; ⊥ Institute of Health Research Gregorio Marañón, Madrid 28007, Spain

**Keywords:** mycobacterium, AMR, MALDI-TOF, robust biomarkers, batch effect, feature selection, machine learning

## Abstract

Distinguishing *Mycobacterium abscessus* subspecies presents significant diagnostic challenges due to their genetic homogeneity and variability in analytical platforms. Our research combines matrix-assisted laser desorption/ionization time-of-flight (MALDI-TOF) mass spectrometry with machine learning (ML) approaches to enhance discrimination accuracy, utilizing 325 spectra profiles from diverse European hospitals. The analytical pipeline incorporates specialized techniques for geographical data harmonization, feature selection, and balancing class representation. The best model employs support vector machines (SVMs) with ComBat correction, Boruta feature selection, and centroid clustering for class imbalance, achieving a discrimination performance of 97% F1 score and 97.17% AUC-ROC on test samples. Noteworthily, most tested models improved their discrimination performance with the approach and demonstrated consistent performance metrics with high geometric mean (GEO) and index balanced accuracy (IBA) metrics (>0.90), ensuring consistent sensitivity and specificity across all subspecies. SHAP (SHapley Additive exPlanations) validated the biological relevance of selected spectral features, particularly improving discrimination of the diagnostically challenging *M. abscessus* subsp. *bolletii* *.* This work advances the state-of-the-art in *M. abscessus* classification, providing a scalable analytical framework for enhanced microbial diagnostics and targeted antimicrobial therapy selection.

## Graphical abstract

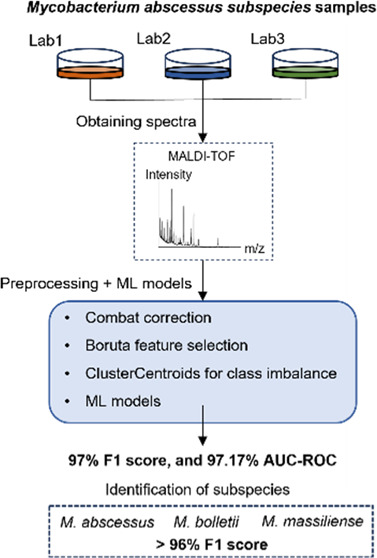

## Introduction

There are many environments in nature in which *Mycobacterium* can be found, including soil, water, and dust. Some of these species are known for their rapid growth and ability to form biofilms in water.^1^ This characteristic allows them to spread and infect both hot- and cold-water drainage systems. Since the start of the 21st century, infections caused by pathogenic bacteria from the nontuberculous mycobacteria group (NTM) have been increasing, leading to higher morbidity and mortality rates.^2,3^

*Mycobacterium abscessus* is a member of the NTM family, and its habitat converges with that of humans. Clinically, it is a major cause of lung and skin infections, particularly in immunocompromised patients.^2^ Moreover, it has emerged as a significant pathogen for individuals with cystic fibrosis, contributing to a global rise in infections.^3^ This fast-growing mycobacterium poses serious challenges in healthcare due to its high antibiotic resistance, often driven by enzyme production that degrades antibiotics or alters their targets.^4,5^

*M. abscessus* is classified into three subspecies that constitute the so-called *M. abscessus* group: *M. abscessus* subsp. *abscessus*, *M. abscessus* subsp. *bolletii*, and *M. abscessus* subsp. *massiliense*, hereafter referred to as *M. abscessus*, *M. bolletii*, and *M. massiliense*, respectively. These three subspecies exhibit significant variation in their antibiotic resistance mechanisms.^6^ For instance, *M. abscessus* and *M. bolletii* are resistant to doxycycline, although the former can sometimes be treated with clarithromycin. In contrast, *M. massiliense* is generally susceptible to both antibiotics.^7^ Due to these differences, identifying the specific subspecies is critical for effective treatment.^8,9^ However, traditional methods often fail to distinguish closely related organisms because they share ribosomal sequences, even though other genes, such as erm(41),^10^ can successfully differentiate them. Despite their higher accuracy, these molecular approaches remain underused due to their higher cost and labor-intensive procedures. In contrast, matrix-assisted laser desorption/ionization time-of-flight mass spectrometry (MALDI-TOF MS)^11^ can identify characteristic proteomic profiles, offering a promising alternative for clinical applications.

MALDI-TOF MS is widely recognized as a rapid and cost-effective tool for identifying bacterial and fungal pathogens due to its ability to analyze proteomic information for accurate, timely analysis.^12^ Despite these strengths, accurately distinguishing among closely related subspecies remains challenging. For instance, conventional MALDI-TOF systems reliably identify the *M. abscessus* complex to the species level (log scores >2.0) but cannot discriminate among subspecies due to highly similar proteomic profiles.^13^ To overcome this limitation, machine learning (ML) techniques have emerged as a promising strategy, with supervised algorithms demonstrating high classification accuracy on sufficiently diverse data sets.^14^ However, many ML models fail to generalize when validation data originate from sources or conditions different from those of the training set, underscoring the need for more robust approaches. Moreover, integrating MALDI-TOF MS data from multiple laboratories often introduces a “batch effect”: systematic variations linked to discrepancies in temperature, humidity, or extraction protocols.^15^ Although batch effect correction techniques are well-established in transcriptomics,^16^ including applications to *Mycobacterium tuberculosis* or *Mycobacterium avium* transcriptomic data,^17,18^ their systematic use in MALDI-TOF MS remains largely unexplored. Addressing the batch effect is critical for enhancing the reliability of ML-based analysis and aligns with new regulations^19^ aimed at improving the efficacy, effectiveness, and efficiency of health technology compared to existing alternatives. Alongside batch effects, antibiotic susceptibility represents a potential confounder that can bias model estimates when resistance is not evenly distributed across subspecies or batches.^20^

MALDI-TOF MS data sets are also often challenging due to their high dimensionality (i.e., thousands of mass-to-charge ratio peaks per spectrum across large numbers of spectra) and significant computational demands. Traditional feature selection methods, such as sequential feature selection, “wrapper” methods, and random forest (RF)-based variable importance, help to mitigate data noise.^21^ However, advanced techniques such as RF-trees are gaining attention for their ability to identify discriminative features.^22,23^ By identifying common and biologically relevant mass peaks across MALDI-TOF MS spectra, advanced feature selection methods can offer innovative preprocessing solutions for differentiating closely related subspecies.^24^ Furthermore, many MALDI-TOF MS data sets suffer from class imbalance, where the minority subspecies receive insufficient representation to train robust predictive models.^25,26^ Resampling methods, such as the synthetic minority oversampling technique (SMOTE),^27^ RandomOverSampler,^28^ ClusterCentroids,^29^ and NeighborhoodCleaningRule,^27^ have proven successful in other domains^30^ but remain underutilized for MALDI-TOF MS data. By integrating batch effect correction, Boruta-based feature selection, and sample bias strategies, approaches that have already demonstrated significant improvements in classification accuracy in transcriptomic data,^31,32^ we believe that these measures can substantially improve such accuracy for MALDI-TOF MS data, enabling more reliable microbial diagnosis. Building on these concepts, we propose an integrated preprocessing pipeline for MALDI-TOF MS data and apply it to a mycobacterium data set collected from nine European hospitals and published in ref 33. Our goal is to develop a robust machine learning classification model capable of accurately distinguishing between *M. abscessus*, *M. bolletii*, and *M. massiliense* *,* thereby serving as a proof of concept for subspecies-level classification of blind samples. This approach addresses key challenges in current MALDI-TOF MS workflows and offers a foundation for broader applications in clinical and research settings.

## Materials and Methods

### Data Sets

Our data set consists of 325 spectra of *Mycobacterium abscessus* samples collected from eight different European hospitals: Hospital General Universitario Gregorio Marañón (HUGM; Madrid, Spain), Hospital Universitario La Princesa (HLP; Madrid, Spain), Instituto de Salud Carlos III-Centro Nacional de Microbiologa (ISCIII; Madrid, Spain), Hospital Universitari de Bellvitge (HUB; Barcelona, Spain), Oslo University Hospital (Oslo, Norway), Brest University Hospital (Brest, France), Radboud University Medical Centre (Nijmegen, The Netherlands), and Sciensano (Brussels, Belgium). A total of 325 spectra were obtained, each corresponding to a distinct strain. For each strain, spectral data were acquired in triplicate across three independent measurements, resulting in nine spectra per strain. These replicates were subsequently averaged during the preprocessing stage to yield a single representative spectrum per strain.

This data set was previously collected at the HUGM and published in ref 33, available at https://zenodo.org/records/17937866. Batch classification labels were assigned based on the city/country of origin of the hospital where the samples were cultured (summary table available in Supporting Information, Table S1). Therefore, we categorized the data into six batches: Madrid (HUGM, HLP, ISCIII), Barcelona, Norway, France, The Netherlands, and Belgium. Additionally, the samples belonged to three *Mycobacterium abscessus* subspecies: *M. abscessus* subsp. *abscessus*, *M. abscessus* subsp. *bolletii*, and *M. abscessus* subsp. *massiliense*, and the number of spectra for each subspecies is 156, 53, and 116, respectively. Among all samples, susceptibility data for clarithromycin (CLA) and amikacin (AMK) were available for 13 isolates, all originating from the HUGM hospital.

### MALDI-TOF Mass Spectra Acquisition, Processing, and Normalization

All isolated samples were incubated at 37 °C for a period of 4 to 7 days. Isolates from HUGM, HLP, and ISCIII, as well as 38 isolates from HUB, were cultured on 7H11 agar plates, whereas the remaining 48 isolates were grown on Löwenstein–Jensen medium, both supplied by BioMérieux, Marcy l’Etoile, France. All isolates were previously identified by PCR-reverse hybridization (GenoType NTM-DR, Hain Lifescience, Nehren, Germany^34^). Protein spectra for HUB samples were acquired on-site at HUB, while spectra for samples from the other hospitals were analyzed at HUGM. Both facilities used a Bruker Daltonics MBT Smart MALDI Biotyper operating over a mass range of 2000–20,000 Da. Further details about extracting the spectra can be found in ref 33.

Spectra were processed, partitioned, aligned, and normalized using the Clover MS Data Analysis Software (Clover MSDAS, provided by Clover Biosoft in Spain).^35^

Preliminary spectra processing involved variance stabilization, accomplished by taking the square root of the intensity values, applying the Savitzky–Golay filter for smoothing (window size of 11, polynomial order of 3), removing the baseline using the Top-Hat filter (factor set at 0.02), and using average spectra calculation for each set of replicates.

Following this, 80% of the data was allocated for model development, while 20% was reserved for testing. Random sampling was ensured through stratification, so each split included enough representative samples from every class. Data partitioning was carried out before alignment and normalization to guarantee the acquisition of separate test spectra rather than a combined matrix. Simultaneously aligning and normalizing the entire data set would have enabled biological information from the test samples to impact the training data. To avert this issue,^36^ we divided the training data and processed each test sample separately. The test samples were aligned and normalized in conjunction with the training matrix, thereby guaranteeing a uniform number of peaks, consistent with the training matrix and each respective test sample. The procedure was then performed for each test sample, leading to all test spectra being preprocessed in relation to the training data while preventing any cross-influence from other test samples.

After the partitioning process, all the spectra were aligned using a common set of peaks by employing a robust point matching approach, which was applied in sequence with a constant tolerance of 2 Da, a linear mass tolerance of 300 ppm, and binned mass spectra measurement points into fixed bins of 0.5 Da, as described in ref 37. The spectra were subsequently normalized by dividing each intensity value in each spectrum by total ion current (TIC), which was calculated as the aggregate sum of all intensity values. The technique is referred to as total ion current (TIC) normalization.^38^

### Harmonization of Geographically Influenced Batch Effects in MALDI-TOF MS Profiles

To address the geographical variation present in MALDI-TOF spectra of the same species cultured in different locations, we employed the ComBat algorithm originally developed for microarray data. ComBat is an open-source tool that offers two correction methods, parametric and nonparametric empirical Bayes approaches, to adjust for batch effects while preserving true biological variation. In this study, we applied the parametric method, which adjusts both location (mean) and scale (variance) effects arising from nonbiological factors. Data normalization was performed prior to applying the ComBat Python package,^39^ as described in the preceding section. The implementation used, pyComBat, is publicly available via the Python Package Index (PyPI) at https://pypi.org/project/combat/, ensuring reproducibility and accessibility. We tested both correction options provided by ComBat: the default method, which adjusts both variance and mean across batches (referred to as TVAR + MEAN), and the mean-only correction, which standardizes the mean while preserving variance (referred to as TMEAN).

On one hand, both ComBat algorithm options were applied to the training partition samples with a geographical batch effect. On the other hand, test samples were first paired with a duplicate of the preprocessed training samples, which still exhibited batch effect. The ComBat algorithm was then run on this combined set. Finally, each test sample was isolated from the training samples after removal of the batch effect. This procedure was performed because the ComBat method needs a data set (as opposed to an individual sample) that encompasses samples from all batches for the purpose of distinguishing between each batch and, consequently, its subsequent adjustment for batch effect. To compare the impact of removing MALDI-TOF MS batches on the performance of machine learning methods, we maintained the original data set, mirroring the current best practices in the field.

The impact of both ComBat removal methods on the control data set was visualized using principal component analysis (PCA)^40^ in conjunction with Clover MSDAS. At this stage, various data sets are extracted from our original data, each with distinct processing methods: (a) control data that has been preprocessed but still contains geographical batch effects; (b) preprocessed data without geographical batch effects corrected using the ComBat method “by var. + mean”; and (c) preprocessed data without geographical batch effects corrected using the ComBat method “by mean”.

### Feature Selection Using Boruta

To reduce the size of the data sets, the Boruta feature selection process was applied (“boruta_py”, an open-source Python implementation of the Boruta R package^41^). This dimensionality reduction method uses random forest (RF) classification to identify the most relevant features. It creates shadow features by randomly shuffling the values of the original features randomly. The algorithm trains the RF model and compares the importance of the original features with those of the shadow features. Features less important than the best shadow feature are considered irrelevant and discarded. This process is repeated iteratively, adjusting the thresholds and verifying the stability of the feature importance. Finally, a set of statistically significant features strongly related to the target variable is obtained.

Feature selection was independently implemented across three distinct training data sets: (1) the raw uncorrected data, (2) data normalized using variance-and-mean batch effect correction, and (3) data processed with mean-only batch effect correction. The resulting subspecies-specific spectral signature lists from each selection process were subsequently applied to their corresponding test and data to ensure methodological consistency. This stratified approach ensures that the data set-specific noise characteristics in the uncorrected data would not contaminate the feature selection process in the batch-corrected data sets, thereby maintaining the integrity of each analytical pipeline. The independent feature selection pathways facilitate a direct comparison of classification performance between correction methodologies while controlling for potential confounding variables.

Statistically, this approach helps to mitigate multicollinearity by selecting a subset of minimally correlated spectral features, enhancing model interpretability while reducing overfitting risk in high-dimensional spectral space. The algorithm preserves the most discriminative variables within each group, capturing the underlying biological signal structure of the data while eliminating redundant information.

### Machine Learning Development Model

For *Mycobacterium abscessus* subspecies classification, we used two state-of-the-art classification algorithms with different capabilities. Random forest (RF)^42^ excels in handling large data sets and providing feature importance and leverages neural network architectures for complex pattern recognition, and support vector machine (SVM)^43^ is effective in high-dimensional spaces and for clear margin separation between classes. We used the scikit-learn package for all models with Python, version 3.11.

Samples separated for the model development were randomly split into a training data set comprising 80% of the samples and a test data set with the remaining 20% while stratifying for the species.

The distribution of the three *Mycobacterium abscessus* subspecies in the samples was imbalanced. “*M. abscessus*” was the most prevalent species, comprising 156 total samples across the batches, followed by *‘*“*M. massiliense”* with 116 samples and “*M. bolletii*” with 53 samples. To address the data set’s imbalance, we employed four different techniques including oversampling and undersampling. RandomOverSampler and SMOTE were the two selected techniques based on oversampling. The two methods based on undersampling were ClusterCentroids and NeighborhoodCleaningRule. All these techniques belong to the imbalanced-learn library,^44^ which is an extension of Python’s Scikit-learn library.

To choose the best model configuration, we determined the optimal hyperparameter set for each model using GridSearch^45^ in conjunction with a 5-fold stratified cross-validation (CV). Using a CV with 5 partitions can provide more accurate estimates of model performance (lower bias), but it requires sufficient data to maintain adequate training partition sizes. By opting for 5-fold CV, we ensured larger training partitions, reducing variance and enhancing the robustness of the performance evaluation.^46,47^ The hyperparameters were tuned to maximize the F1-weighted score, evaluated exclusively on the training data set of the corrected data, to obtain a comparison with the uncorrected data. The process was identical with the two data sets “no_batch_effect_by_default” and “no_batch_effect_by_mean”.

Given that antibiotic susceptibility can confound model training and evaluation,^20^ we examined the distribution of the 13 samples with recorded susceptibility to clarithromycin (CLA) and amikacin (AMK) across the 5-fold cross-validation partitions. We also conducted PCA to explore potential clustering by resistance phenotype in the spectral space, as such a structure could influence classification performance.

### Evaluation Metrics

After determining the optimal hyperparameters, model fitting was performed to estimate the final parameters, with the best-performing model selected based on its evaluation metrics. The evaluation framework included standard performance measures, such as accuracy, precision, recall, F1 score, area under the receiver operating characteristic curve (AUC-ROC), and area under the precision-recall curve (AUC-PRC). To address the challenges of imbalanced classification, additional metrics specifically designed for such scenarios, including balanced accuracy,^48^ the geometric mean score (GEO),^49^ and the Index of Balanced Accuracy (IBA),^30^ were utilized.

### Explainability for Understanding Strain Differences in MALDI-TOF Classification

To gain insights into strain-specific differences identified by MALDI-TOF MS mass spectrometry classifiers, we employed SHAP (SHapley Additive exPlanations), a powerful tool for interpreting machine learning models. SHAP values provided a quantitative measure of how individual features influenced the model’s predictions, facilitating both global insights into features driving strain differentiation and local insights into the unique characteristics of specific strains. Summary plots were generated to visualize global feature importance across strains and local contributions for individual predictions, highlighting the key spectral patterns associated with strain-level variations.

### Complete Workflow

As illustrated in Figure 1, the methodological pipeline begins with (a) data collection, where MALDI-TOF MS spectra were obtained from multiple collaborating hospitals. Detailed metadata regarding sample origin and acquisition dates are provided in the corresponding section.

**Figure 1:**
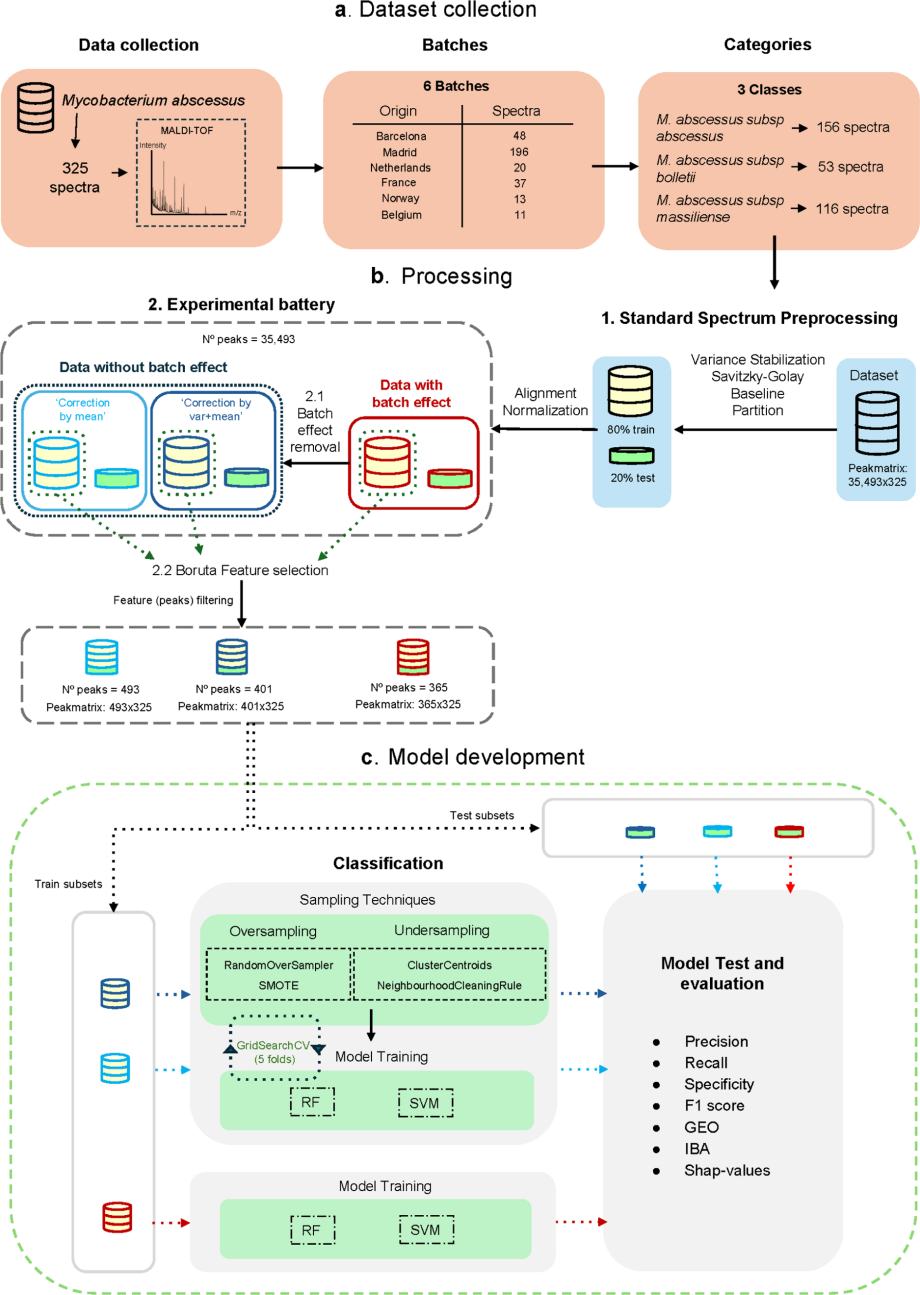
Overview of the methodological workflow. This schematic outlines the complete analytical pipeline used for MALDI-TOF MS data classification. The workflow includes the following: (a) data collection from multiple hospitals; (b) spectral preprocessing, data set partitioning, and batch effect correction using two methods (“by var. + mean” and “by mean”); (b.1) experimental battery creation with three branches (no correction, var. + mean, and mean); (b.2) feature selection using Boruta applied to each branch; (c) model development, including oversampling and undersampling for class balance, followed by independent training and validation of classifiers. Each step is labeled to facilitate cross-referencing with the detailed explanation provided in the “Complete Workflow” section of Materials and Methods, techniques, and the development of classification models for MALDI-TOF MS data analysis.

The workflow then proceeds to (b) processing, which includes several preprocessing steps: variance stabilization, smoothing, baseline subtraction, and averaging of replicate spectra. The resulting data set is partitioned into 80% for training and 20% for testing. To ensure consistency in peak alignment, both partitions undergo normalization. Specifically, the training set is normalized independently, while each test sample is appended to a copy of the training set and jointly aligned and normalized to ensure a consistent peak structure across both sets. Following preprocessing, the experimental battery is constructed by splitting the processed data into three branches: no batch effect correction; batch correction using “by var. + mean”; and batch correction using “by mean”. These corrections are detailed in Section 2.1 Batch Effect Removal Experimental Battery. Feature selection is performed using the Boruta algorithm (Section 2.2), applied independently to each of the three training subsets. The resulting peak lists are then transferred to their corresponding test partitions to ensure a consistent feature representation. The workflow then advances to (c) model development, where machine learning classifiers are trained separately on each of the three training subsets. For the two batch-corrected data sets, oversampling and undersampling techniques are applied to address class imbalance. The uncorrected data set proceeds directly to model training without resampling. Each trained model is subsequently validated by using its corresponding test partition.

This structured approach ensures that the impact of batch correction and class balancing on model performance is rigorously evaluated, supporting the robustness and generalizability of the classification framework.

## Results

### Evaluation of ComBat as a Geographical Harmonization Method

Because every isolate was run on the same Bruker MBT Smart MALDI-TOF MS with identical acquisition settings at both Madrid and Barcelona, analyzing samples at two locations introduced no platform-related batch effect, a conclusion supported by the absence of site-specific clustering.

However, principal component analysis (PCA) of the processed, aligned, and normalized data set revealed a clear geographical batch effect, with samples clustering according to their hospital of origin rather than forming a uniform distribution (Figure 2). This segregation likely stems from variations in culture conditions including incubation temperature, humidity, media handling, and other laboratory-specific parameters that precede spectral acquisition across different facilities. Such geographically driven shifts can substantially compromise the performance of machine learning models, necessitating effective batch correction strategies.

**Figure 2:**
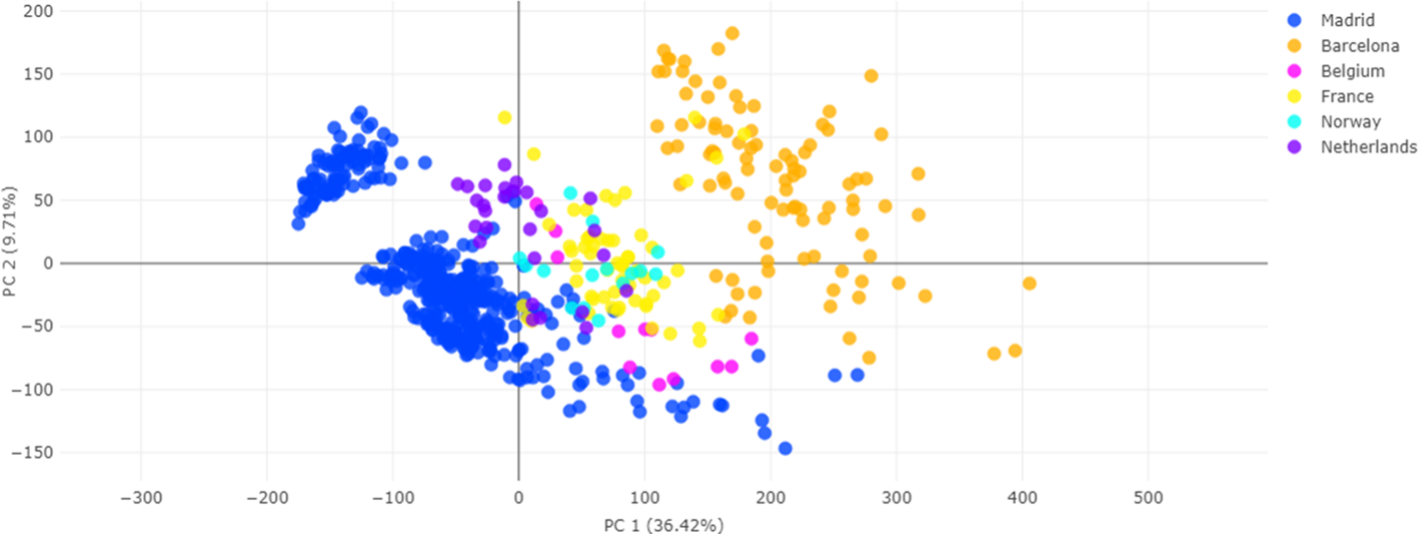
Principal component analysis (PCA) on the training partition data, illustrating the batch effect. The plot displays the two principal components, with data points color-coded by the hospital of origin (batch). Each color represents one or more contributing hospitals.

To address this challenge, we implemented the ComBat algorithm to this processed, aligned, and normalized data set using two distinct methodologies: “var. + mean” and “mean”. Both approaches effectively mitigated batch effects (Figures 3 and S1), although the “var. + mean” methodology demonstrated superior performance with this data set (Figure 3). Postcorrection analysis revealed a more homogeneous sample distribution, significantly enhancing data consistency across geographical origins. Detailed descriptions of the “mean” batch correction methodology and its corresponding visualizations are provided in the Supporting Information (Figures S1 and S2). In both correction approaches, the ComBat application resulted in markedly improved uniformity of the sample distribution compared to the uncorrected data.

**Figure 3:**
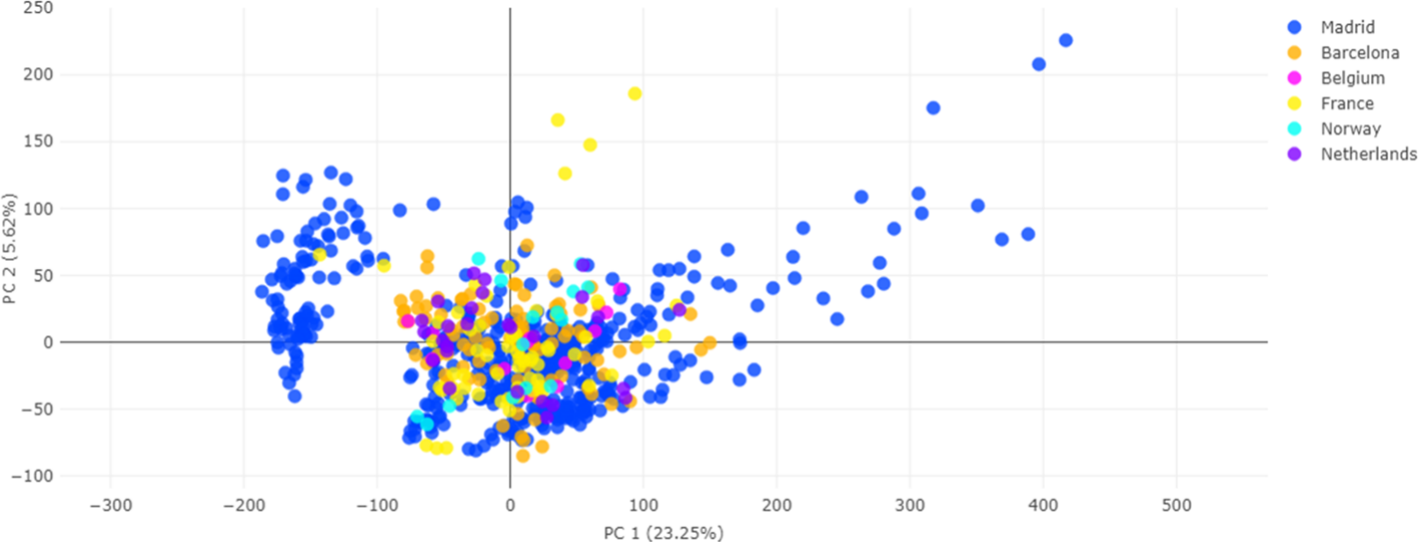
Principal component analysis (PCA) of the training data set after “var. + mean” batch effect correction. The plot displays the first two principal components, with data points color-coded by the hospital of origin (batch). Each color represents one or more contributing hospitals.

Spectral analyses confirmed the presence of pronounced batch effects prior to correction, particularly within the 5700–5800 *m*/*z* range, where peak alignment varied markedly among batches (Figure 4). After applying the “var. + mean” (Figure 5), these discrepancies were notably reduced, and interbatch spectra displayed improved alignment and intensity standardization. For instance, the previously subtle 5650 *m*/*z* peak observed in the Madrid batch became clearly defined, while other batch-specific peaks converged toward consistent intensity levels. Collectively, these results demonstrate the superior performance of the “var. + mean” approach in mitigating geographical batch effects in this data set. Complementary results for the “mean” correction approach are presented in the Supporting Information (Figure S2).

**Figure 4:**
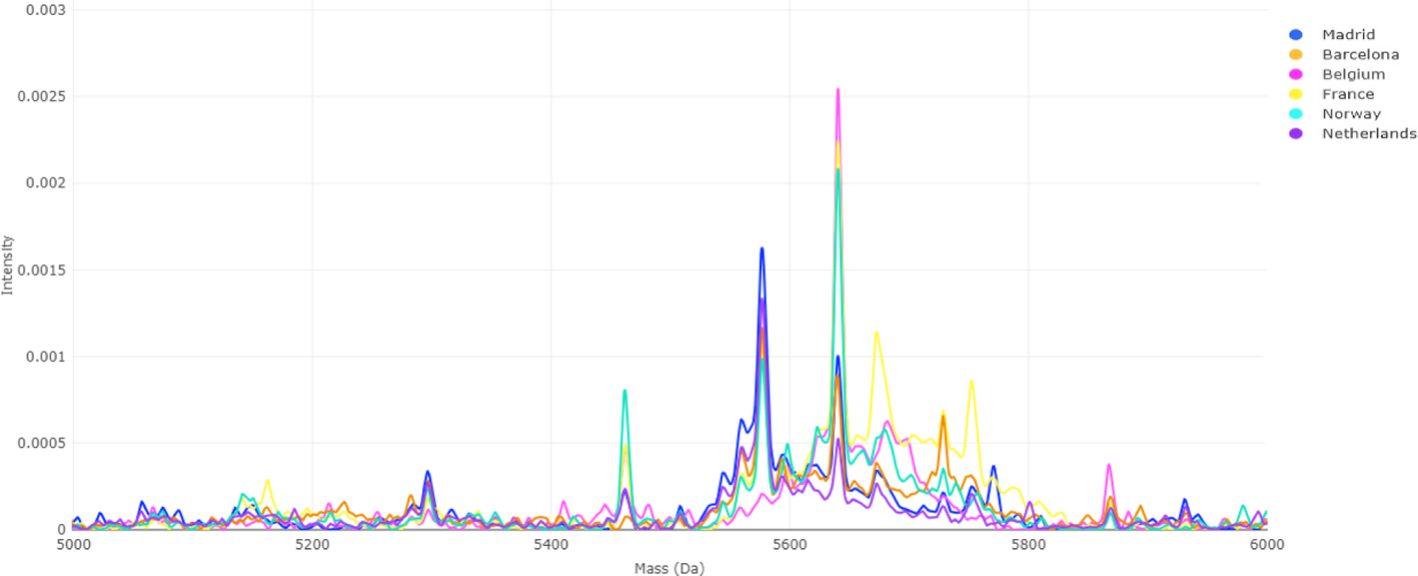
MALDI-TOF MS spectra before batch effect correction. Color-coded spectra represent isolates from six hospitals (Madrid, Barcelona, Belgium, France, Norway, and The Netherlands). Interbatch variability is visible as peak intensity and alignment differences across the 5700–5800 *m*/*z* region, reflecting technical rather than biological variation.

**Figure 5:**
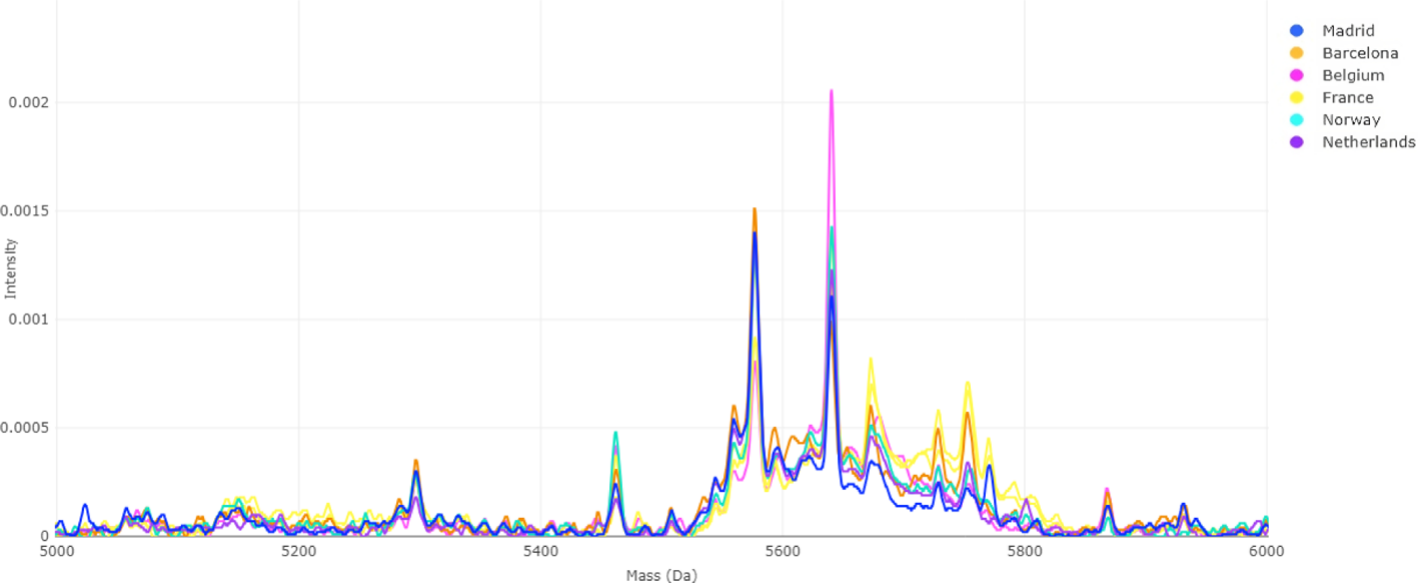
MALDI-TOF MS spectra after “var. + mean” batch effect correction. Spectra corresponding to the same samples shown in Figure 4 after application of the “var. + mean” ComBat correction. The improved alignment and uniform peak intensities indicate effective reduction of interbatch variability while preserving biological consistency.

### Feature Selection

The Boruta algorithm was employed for feature selection to identify the most informative mass-to-charge (*m*/*z*) peaks from the original data set. This process substantially reduced the feature space from 35,493 initial mass peaks across all 325 MALDI-TOF MS spectra to three distinct subsets: 365 peaks for uncorrected spectra, 401 peaks following “by var. + mean” batch correction, and 493 peaks after “by mean” batch correction. The feature reduction achieved dimensionality reductions of 98.97%, 98.87%, and 98.61%, respectively, thereby mitigating the curse of dimensionality while retaining the most discriminative spectral features for subsequent machine learning analysis. The complete list of selected peaks for each subset is provided in Supporting Information Table S2.

### Impact of Geographical Harmonization, Feature Selection, and Sample Balancing Techniques on the Development of ML Models

To systematically evaluate the influence of geographical harmonization (batch effect correction), feature selection, and sample balancing techniques on MALDI-TOF MS-based Mycobacterium subspecies classification, we assessed two machine learning algorithms: random forest (RF) and support vector machine (SVM). Our experimental framework comprised three distinct analytical conditions: a baseline scenario without batch correction, feature selection, or resampling, and two approaches incorporating ComBat batch correction methodologies (“var. + ” and “mean”) integrated with Boruta-based feature selection. We further implemented four resampling strategies to address class imbalance challenges: RandomOverSampler, SMOTE, ClusterCentroids, and NeighborhoodCleaningRule. Result tables present the classification performance metrics for optimally parametrized models, with ClusterCentroids emerging as the superior resampling methodology. Comprehensive results for alternative preprocessing combinations are provided in Supporting Information (Tables S4 and S5), while the optimized hyperparameter configurations for each classifier are detailed in Supporting Information Table S3.

#### Performance without Geographical Harmonization

In the absence of batch effect correction (Tables 1 and 2), random forest (RF) demonstrated superior performance with an average F1 score of 0.86 and balanced accuracy of 0.84, compared to support vector machine (SVM) with values of 0.80 and 0.79, respectively. RF consistently outperformed SVM across all key performance metrics under these uncorrected conditions. However, the presence of substantial geographical batch effects evidently constrained the overall classification potential of both algorithms, emphasizing the need for effective correction strategies.

**Table 1:** Performance Metrics for Classification Models on Test Data without Batch Effect Correction^a^

Models	Class	Precission	Recall	Specificity	F1	GEO	IBA	Support
RF	*M. abscessus*	0.85	0.90	0.85	0.88	0.88	0.77	31
	*M. bolletii*	1.00	0.80	1.00	0.89	0.89	0.78	10
	*M. massiliense*	0.83	0.83	0.90	0.83	0.86	0.74	23
SVM	*M. abscessus*	0.79	0.87	0.79	0.83	0.83	0.69	31
	*M. bolletii*	0.73	0.80	0.94	0.76	0.87	0.74	10
	*M. massiliense*	0.84	0.70	0.93	0.76	0.80	0.63	23

a The number of samples for each subspecies is displayed in the sup column.

**Table 2:** Average Performance Metrics for Classification Models on Test Data without Batch Effect Correction

avg/models	RF	SVM
avg_pre	0.86	0.80
avg_rec	0.86	0.80
avg_spe	0.89	0.86
avg_f1	0.86	0.80
avg_geo	0.87	0.83
avg_iba	0.76	0.68
accuracy	0.86	0.80
balanced accuracy	0.84	0.79
AUC	0.97	0.93
total_support	64	64

#### Performance of “var. + mean” Geographical Harmonization

Implementation of the “var. + mean” correction methodology yielded substantial performance enhancements across all classification models, with the support vector machine (SVM) demonstrating a greater improvement. The SVM classifier exhibited a remarkable 17% increase in F1 score relative to uncorrected data, while random forest (RF) also showed significant performance gains (Tables 3 and 4). The optimized SVM achieved exceptional discriminative capability with 97% accuracy in test samples, surpassing previously established benchmarks in the literature.^33^ Particularly noteworthy was the improved classification of *M. abscessus* subsp. *bolletii*, historically the most challenging subspecies to identify accurately, which demonstrated marked improvement across all modeling approaches. Following geographic harmonization, model performance converged, with RF and SVM classifiers achieving comparable accuracy (0.92–0.97) and area under the curve (AUC) values (0.97–0.98). These findings underscore the critical importance of geographical harmonization techniques in mitigating interdata set variability and enhancing classification robustness. Comprehensive evaluation metrics, including weighted precision, recall, and F1 scores, are detailed in Supporting Information Table S4.

**Table 3:** Performance Metrics by Class for Classification Models on Test Data Using “var. + mean” Batch Effect Correction, Boruta Feature Selection, and the ClusterCentroids Sampling Technique^a^

Models	Class	Precission	Recall	Specificity	F1	GEO	IBA	Support
RF	*M. abscessus*	0.96	0.87	0.97	0.92	0.92	0.84	31
	*M. bolletii*	0.83	1.00	0.96	0.91	0.98	0.97	10
	*M. massiliense*	0.92	0.96	0.95	0.94	0.95	0.91	23
SVM	*M. abscessus*	0.97	0.97	0.97	0.97	0.97	0.94	31
	*M. bolletii*	1.00	1.00	1.00	1.00	1.00	1.00	10
	*M. massiliense*	0.96	0.96	0.98	0.96	0.97	0.93	23

a The number of samples for each subspecies is displayed in the sup column.

**Table 4:** Average Performance Metrics for Classification Models on Test Data Using “var. + mean” Batch Effect Correction, Boruta Feature Selection, and the ClusterCentroids Sampling Technique

avg/models	RF	SVM
avg_pre	0.93	0.97
avg_rec	0.92	0.97
avg_spe	0.96	0.98
avg_f1	0.92	0.97
avg_geo	0.94	0.97
avg_iba	0.88	0.95
accuracy	0.92	0.97
balanced accuracy	0.94	0.97
AUC	0.98	0.97
total_support	64	64

#### Performance of “Mean” Geographical Harmonization

Implementation of the “mean” batch correction methodology yielded performance enhancements comparable to the “var. + mean” approach. The support vector machine (SVM) classifier demonstrated exceptional discriminative capability, achieving 97% accuracy with corresponding weighted precision, recall, and F1 scores, along with an area under the receiver operating characteristic curve (AUC-ROC) of 98.30% in test samples (Tables 5 and 6, Supporting Information Table S5). The random forest (RF) algorithm exhibited slightly diminished performance metrics relative to SVM, mirroring the pattern observed with the “var. + mean” correction approach.

**Table 5:** Average Performance Metrics for Classification Models on Test Data Using “mean” Batch Effect Correction, Boruta Feature Selection, and the ClusterCentroids Sampling Technique^a^

Models	Class	Precission	Recall	Specificity	F1	GEO	IBA	SUpport
RF	*M. abscessus*	0.97	0.90	0.97	0.93	0.94	0.87	31
	*M. bolletii*	0.77	1.00	0.94	0.87	0.97	0.95	10
	*M. massiliense*	0.95	0.91	0.98	0.93	0.94	0.89	23
SVM	*M. abscessus*	0.97	0.97	0.97	0.97	0.97	0.94	31
	*M. bolletii*	0.91	1.00	0.98	0.95	0.99	0.98	10
	*M. massiliense*	1.00	0.96	1.00	0.98	0.98	0.95	23

a The number of samples for each subspecies is displayed in the sup column.

**Table 6:** Average Performance Metrics for Classification Models on Test Data Using “mean” Batch Effect Correction, Boruta Feature Selection, and the Cluster Centroids Sampling Technique

avg/models	RF	SVM
avg_pre	0.93	0.97
avg_rec	0.92	0.97
avg_spe	0.97	0.98
avg_f1	0.92	0.97
avg_geo	0.94	0.98
avg_iba	0.89	0.95
accuracy	0.92	0.97
balanced accuracy	0.94	0.97
AUC	0.97	0.98
total_support	64	64

All harmonization-enhanced models substantially outperformed their uncorrected counterparts across evaluation metrics. Notably, both SVM and RF algorithms, when integrated with either “var. + mean” or “mean” correction methodologies, consistently achieved superior classification accuracy for all subspecies classifications, surpassing the previously established performance benchmarks in the literature.^33^

#### Impact of Sample Balancing Techniques

Among the diverse resampling methodologies evaluated, ClusterCentroids demonstrated superior classification performance, particularly when integrated with the support vector machine (SVM) algorithm, achieving an exceptional F1 score of 97% in independent test samples. NeighborhoodCleaningRule, RandomOverSampler, and SMOTE also exhibited robust classification capabilities when implemented in conjunction with the mean batch correction methodology and SVM classifier. However, these three resampling algorithms delivered more moderate performance improvements when paired with the “var. + mean” batch correction approach, generally surpassing uncorrected data performance metrics but not reaching the classification efficacy achieved by ClusterCentroids (Supporting Information Table S4). Collectively, these findings emphasize the critical importance of implementing both effective geographical harmonization and appropriate sample balancing strategies to optimize machine learning-driven subspecies classification using MALDI-TOF MS spectral data.

#### Models’ Explainability

The interpretability of the optimal support vector machine (SVM) classifier was assessed using the SHapley Additive exPlanations (SHAP) methodology (Figure 6), which decomposes each prediction into individual feature contributions. This analytical framework provides comprehensive global and local interpretability, quantifying the relative importance of each spectral feature in subspecies classification while offering insights into how specific spectral peaks influence model predictions. The ranked significance of the top 27 peaks clearly delineates their proportional contribution to classification outcomes, highlighting key discriminative driving model performance.

**Figure 6:**
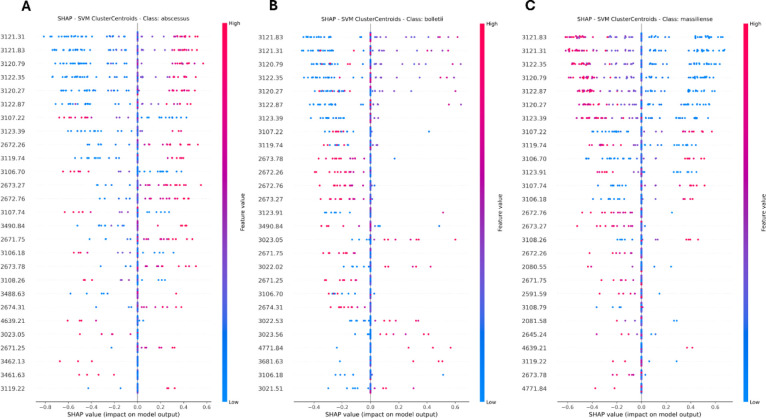
SHAP summary plot for the best-performing model (SVM) with “var. + mean” batch effect correction. This figure presents SHAP value analysis for the SVM model, illustrating feature importance and impact on prediction. Each row represents a peak, with points color-coded by peak values (red for high and blue for low). Points positioned further to the right contribute positively to the model’s prediction, while those to the left have a negative impact. (A) SHAP summary plot for *M. abscessus* subsp. *abscessus*, (B) SHAP summary plot for *M. abscessus* subsp. *bolletii*, and (C) SHAP summary plot for *M. abscessus* subsp. *massiliense*.

Figures 6A, 6B, and 6C illustrate SHAP summary plots, where individual rows represent specific spectral peaks, and data points correspond to individual samples within the data set. The color gradient indicates feature intensity (red denoting high intensity and blue indicating low intensity), while horizontal displacement indicates the feature’s contribution to model predictions for subspecies-specific classification decisions. These visual representations enhance model interpretability by revealing the directional and magnitude-specific influences of each peak on algorithmic outcomes. SHAP analysis of the best-performing SVM model, harmonized using the “var. + mean” batch correction method (Table S6), revealed distinct subspecies-specific peak signatures with characteristic overlap patterns: *M. bolletii* and *M. massiliense* shared 15 discriminative peaks, whereas *M. abscessus* shared up to 22 peaks with *M. massiliense* (Figure S3). A comparable distribution pattern was observed in the RandomOverSampler + RF model under identical batch correction parameters (Table S7), where 8–10 of the top 27 peaks overlapped between *M. abscessus* and *M. massiliense*, contrasting with fewer than 5 overlaps with *M. bolletii*. These differential overlap patterns align with established phylogenetic relationships between subspecies and demonstrate the model’s capacity to capture biologically relevant signatures while mitigating the geographical batch effect. Supporting Information Figures S3–S7 provide an extended analysis of peak commonality across different preprocessing methodologies and subspecies.

All evaluated models implementing our integrated approach demonstrated substantial improvements in classifying the most challenging subspecies, *M. bolletii*, consistently outperforming previously published approaches across all classifiers. The high classification accuracy was maintained across multistrain samples, indicating strong generalization and robustness. The highest-performing models consistently achieved geographic and imbalanced accuracy (GEO and IBA) scores exceeding 0.95, reflecting well-balanced classification performance across all subspecies, including those with limited representation in the training data set. These results underscore the efficacy of our comprehensive machine learning pipeline in addressing class imbalance while maintaining superior predictive accuracy, particularly for taxonomically challenging subspecies.

#### Susceptibility to CLA and AMK Resistance

Susceptibility data for clarithromycin (CLA) and amikacin (AMK) were available for only 13 isolates (4% of the data set; 13/325), all from HUGM, precluding inclusion of resistance as a modeling variable. PCA of these isolates (Supporting Information Figure S8) showed clustering driven by subspecies identity rather than resistance phenotype: resistant and susceptible isolates colocalized within the same subspecies clusters, including within *M. massiliense* across multiple resistance profiles (AMK_R_CLA_S, AMK_S_CLA_R, AMK_S_CLA_S), and similarly within *M. abscessus* subsp. *abscessus* and *M. bolletii*. We also examined the 5-fold cross-validation split used for modeling (seed = 42; Supporting Information Table S8) and confirmed that the 13 annotated isolates were distributed across multiple folds (e.g., 4 in fold 0 and 1 in fold 1), with none concentrated in a single fold. Together, these results indicate that the dominant structure in the data reflects subspecies-specific proteomic profiles, not antibiotic resistance, and that susceptibility-annotated isolates are inherently dispersed across the cross-validation partitions.

### Discussion

Traditional methods for identifying *Mycobacterium abscessus* subspecies are now increasingly enhanced by machine learning (ML) techniques,^50^ which utilize the rich proteomic data provided by MALDI-TOF MS spectra.^33^ Accurate subspecies identification has significant clinical implications as different subspecies demonstrate varying antibiotic resistance profiles and treatment outcomes. However, the reliability of these ML classifiers can be affected by several challenges, including batch effects, high dimensionality, and class imbalance. In this study, we systematically addressed each of these issues, achieving notable gains in subspecies classification performance.

Our data set, which comprises samples of three *M. abscessus* subspecies collected from six regions (Madrid, Barcelona, Norway, France, The Netherlands, and Belgium), presented substantial variability. This heterogeneity reflects environmental differences (e.g., water, food, and habits) that could drive microevolutionary events, a concept well documented in gut microbiota studies.^51^ Accordingly, we constructed training partitions to ensure that models were exposed to a broad spectrum of geographic samples, thereby enhancing their capacity for generalization across divergent laboratory conditions.

#### Geographical Harmonization

Initial PCA analysis highlighted pronounced geographical batch effects, evident in the clustering of samples by laboratory rather than subspecies (Figure 2). This segregation likely stems from variations in culture conditions including temperature, humidity, and other laboratory-specific parameters across different facilities.^15^ Such geographical stratification can substantially compromise the performance of machine learning models, necessitating effective batch correction strategies. Peak alignment inconsistencies in the 5700–5800 Da range (Figure 4) underscored the severity of this issue.

Processing every sample on identically configured MALDI-TOF MS instruments operated under harmonized parameters in both Madrid and Barcelona effectively eliminated platform-related variability, and we could attribute the remaining clustering exclusively to the geographic origin. This controlled setting allowed us to isolate and evaluate the specific impact of geographical sample origin on MALDI-TOF MS results with greater precision than previous multi-instrument studies.

By applying the ComBat algorithm with both “var. + ” and “mean” strategies, we substantially harmonized the data sets without erasing critical phenotypic markers (Figures 3 and 5). We selected ComBat based on its demonstrated success in transcriptomics and proteomics studies, where it has shown remarkable ability to preserve biological signal integrity while effectively removing technical variations.^39^ The “var. + m” approach showed superior performance, particularly for smaller batches, while the “mean” strategy occasionally helped avoid overcorrection in larger data sets (Figures S1 and S2). That our implementation of ComBat for MALDI-TOF MS successfully reconciled proteomic data further underscores its versatility in high-dimensional classification tasks.

Despite these improvements, a potential limitation is that batch effect correction might overfit specific laboratory conditions if the data set is not sufficiently diverse or if certain laboratories contribute with very few samples. Future studies could explore alternative correction algorithms (e.g., RUV-based methods) or apply cross-laboratory validation strategies to verify the robustness of the corrected models.

#### Dimensionality and Feature Selection

MALDI-TOF MS data are notoriously high-dimensional, often including tens of thousands of peaks,^52^ presenting computational challenges for both hyperparameter optimization and routine model training. Using Boruta, we efficiently condensed the data set from 35,493 peaks to 401 or 493 (for “var. + mean” and “mean” corrections, respectively). This reduction retained critical discriminatory features while minimizing the computational overhead.

Boruta demonstrated strong alignment with the study’s objectives, streamlining the data set for subsequent processing steps such as batch effect correction. This reduction significantly lowers computational demands, facilitating faster model training and efficient hyperparameter tuning. Indeed, Boruta proved well suited to preserving subtle intersubspecific differences, enabling faster and more robust classifier performance.^53^ Although our results confirm Boruta’s utility for a range of classification algorithms, future work might still investigate alternative feature selection strategies or neural network-based embeddings. Such methods could further refine feature representation, particularly for highly heterogeneous or larger-scale data sets, thus augmenting the robust performance already observed in this study.

#### Class Imbalance and Model Performance

Class imbalance was particularly pronounced in samples of *M. abscessus* subsp. *bolletii*, especially from certain geographical origins (e.g., Norwegian and Madrid HLP batches, which lacked this subspecies entirely). Imbalanced data sets can bias ML models,^54^ potentially inflating performance metrics for majority classes while compromising the detection of minority classes.

Among the tested resampling techniques, ClusterCentroids and RandomOverSampler consistently yielded more balanced and accurate predictions. This method performed particularly well because it addressed the class imbalance problem by reducing the majority of classes while preserving their structural diversity and informational content. Importantly, its use significantly boosted the recognition of *M. bolletii*, a historically under-detected subspecies.^33^ RandomOverSampler also demonstrated a strong performance by equalizing class distributions without information loss. These findings highlight the synergy between batch effect correction and judicious resampling as methods for improving classifier reliability. Nevertheless, the extent to which these resampling strategies replicate the true biological diversity of real-world samples remains an open question, warranting further study with larger or more diverse data sets.

#### Classifier Comparison

Among the evaluated algorithms, support vector machine (SVM) and random forest (RF) demonstrated the strongest performance, achieving F1 scores of 94–97% and area under the ROC curve (AUC-ROC) values of 97% in the test samples. Notably, these metrics were independent of the samples’ geographic origin, underscoring the generalizability of our approach. This level of accuracy surpasses the previously reported 88.6% by Rodríguez-Temporal et al. (2023) for multigeographical samples, establishing a new benchmark for *M. abscessus* subspecies classification. Importantly, high GEO and IBA metrics (>0.90) confirmed the balanced success of these models, ensuring that both sensitivity and specificity were optimized across all three subspecies, even the most challenging *M. bolletii*.

Despite these promising results, the risk of overfitting still remains. While cross-validation and hyperparameter tuning were carefully conducted, prospective external validations or clinical trials could further solidify confidence in the real-world reliability of these models. Additionally, more granular clinical data, such as patient outcomes and antibiotic resistance profiles, may bolster the translational utility of the findings.

#### Model Interpretability

To facilitate clinical adoption, model interpretability is essential. Our SHAP value analysis showed that feature selection was refined by var. + mean correction, aligning with biologically relevant peaks. The analysis revealed three primary discriminative *m*/*z* regions (∼2672, ∼3105, and ∼3120), with multiple closely spaced features within each region reflecting different sampling points of the same underlying spectral peak rather than distinct biological entities. This pattern is expected in linear-mode MALDI-TOF MS, where small interrun shifts and sampling of adjacent *m*/*z* points can distribute feature importance across neighboring positions. Given the subtle differences inherent to subspecies-level discrimination, particularly the historical under-detection of *M. abscessus* subsp. *bolletii*, we intentionally preserved fine spectral structure through 0.5 Da binning to capture small, within-peak variations in shape, shoulders, and local intensity ratios (see Supporting Information Figure S9). Multiple data points within each peak profile (e.g., peaks 3121.31 and 3121.83 representing different positions within the same biological signal, Supporting Information Table S6) provide complementary information about peak morphology that would be lost with coarser binning approaches. This fine-resolution strategy enabled our model to achieve balanced performance across subspecies, with F1 scores for *bolletii* (0.89) approaching those of the other subspecies. The identified *m*/*z* regions likely correspond to abundant protein signals typical of MALDI-TOF microbial fingerprints; however, without targeted identification, we present them conservatively as discriminative *m*/*z* regions rather than assigned proteins. This approach demonstrates that subspecies discrimination depends not only on peak presence/absence but also on subtle differences in peak morphology and intensity profiles within critical mass ranges, differences that require fine-scale feature resolution to capture effectively.

#### Limitations and Future Directions

Although our integrated pipeline markedly improved subspecies classification, several limitations need consideration. First, the generalizability of these techniques to other bacteria or fungal pathogens remains untested and represents an important avenue for future work. Second, the analysis was confined to samples from nine European hospitals; global validation with broader geographic diversity could reveal new sources of batch effects or differences in subspecies prevalence.

Although processing every specimen on identically configured MALDI-TOF MS instruments at both hospitals effectively removed platform-related variation in this study, real-world workflows will inevitably involve a mix of instrument models and firmware versions across laboratories. Future studies should explore how our harmonization techniques perform when instrumental variations are also present.

Future research could explore whether the spectral features that enable subspecies differentiation also capture molecular signatures associated with antimicrobial resistance patterns, providing rapid and complementary insights into traditional antibiotic susceptibility testing methods. However, this would require larger, balanced data sets that integrate both high-quality spectra and comprehensive resistance phenotypes.

## Conclusion

By simultaneously addressing geographical batch effects, high dimensionality, and class imbalance, we improved the robustness and accuracy of MALDI-TOF MS-based classification models for the *M. abscessus* subspecies. Our pipeline, comprising ComBat correction, Boruta-based feature selection, and strategic resampling, sets a new performance benchmark, particularly for the challenging *M. bolletii*. These results highlight the potential of advanced machine learning frameworks in clinical microbiology and contribute to ongoing efforts aimed at harmonizing multilaboratory MALDI-TOF MS data.

In future applications, this integrated approach could be delivered as modular scripts or software packages to enable streamlined and semiautomated spectral analysis. Such tools could be embedded in routine laboratory workflows to reduce manual intervention and improve reproducibility. With the growing availability of automated preprocessing libraries and increasingly seamless instrument software integration, near-real-time subspecies detection from raw spectra may be feasible in well-standardized laboratory environments with adequate computational resources.

From a technological standpoint, machine learning in clinical microbiology is advancing rapidly. Deep learning models, particularly convolutional neural networks (CNNs), are being explored for spectral pattern recognition in MALDI-TOF MS. Their broader adoption remains constrained by the need for large, well-curated data sets to prevent overfitting, interinstrument/site variability that can limit generalizability, and the black-box nature of complex models, which challenges clinic interpretability. Priorities to address these barriers include explainable AI (XAI) to improve transparency, standardized preprocessing, and quality control across laboratories, and practical, user-friendly platforms that couple end-to-end ML pipelines with MALDI-TOF instruments; hardware acceleration (e.g., GPU-based processing) may further reduce runtimes and support near real-time analysis.

Altogether, this study offers a scalable and adaptable framework that links technical innovation with clinical applicability; nevertheless, these findings should be interpreted with appropriate caution and would benefit from independent, multicenter, prospective validation to establish robustness and clinical impact across diverse settings.

## Supplementary Material





## Data Availability

The Mycobacterium data set analyzed in this study is currently available in the following public repository: https://zenodo.org/records/17937866.

## Notes

Principal component analysis (PCA) of training data after “mean-based” batch effect correction, highlighting two principal components; spectrum plots after ‘mean-based’ batch effect correction; common spectral peaks among *M. abscessus* subspecies after “var. + mean” batch correction and ClusterCentroids resampling and SVM; common spectral peaks among *M. abscessus* subspecies after “var. + mean” batch correction and RandomOverSampler resampling and RF; common spectral peaks among ClusterCentroids resampling with SVM and ROS resampling with RF after “var. + mean” batch correction for *M. abscessus* subspecies; common spectral peaks among ClusterCentroids resampling with SVM and ROS resampling with RF after “var. + mean” batch correction for *M. bolletii* subspecies; common spectral peaks among ClusterCentroids resampling with SVM and ROS resampling with RF after “var. + mean” batch correction for *M. massiliense* subspecies; principal component analysis (PCA) analysis of antibiotic resistance profiles in *M. abscessus* subspecies; and average intensities per *M. abscessus* subspecies with standard deviation for primary discriminative *m*/*z* regions (PDF)
Data set summary table; list of selected peaks by subset using Boruta; hyperparameters: tested and selected configurations for the classification algorithms; performance metrics (and average performance) of the classification models on the test data using “var. + mean” batch effect correction, Boruta feature selection, and different sampling techniques; performance metrics (and average performance) of the classification models on the test data using “mean” batch effect correction, Boruta feature selection, and different sampling techniques; top feature intersections between *Mycobacterium abscessus* subspecies with “var. + mean” correction and the combination of ClusterCentroids resampling and SVM; comparison of top peaks between the two best combinations of resampling methods and classification models; and real split reproduction for 13 isolates with available antibiotic susceptibility (XLSX)

## Notes

Ethical Guidelines: According to the published studies, all were performed with proper ethics.

## Abbreviations

NTM: nontuberculous mycobacteria
MALDI-TOF MS: matrix-assisted laser desorption/ionization time-of-flight mass spectrometry
ML: machine learning
SMOTE: synthetic minority oversampling technique
HUGM: Hospital General Universitario Gregorio
HLP: Hospital Universitario La Princesa
ISCIII: Instituto de Salud Carlos III
HUB: Hospital Universitari de Bellvitge
CLA: Claritromicina
AMK: Amikacina
Clover MSDAS: Clover MS Data Analysis Software
TIC: total ion current
PCA: principal component analysis
RF: random forest
SVM: support vector machine
CV: cross-validation
AUC-ROC: area under the receiver operating characteristic curve
AUC-PRC: area under the precision-recall curve
GEO: geometric mean score
IBA: index of balanced accuracy.
