# Supplementary material for: Toward Robust Machine Learning Models for MALDI-TOF MS: Novel Approaches for Mycobacterium abscessus Subspecies Identification
Source: J Proteome Res. 2026 Feb 9;25(3):1305–18. doi: 10.1021/acs.jproteome.5c00534 (PMC12973297; doi:10.1021/acs.jproteome.5c00534)

Towards Robust Machine Learning Models for MALDI-TOF MS: Novel Approaches for *Mycobacterium abscessus* Subspecies Identification

Erica Padial-Fuillerat [1,2,3]‡, *Juan E. Martínez-Manjón* [2,3]‡, *Igor Zwir* [2,3], *Manuel J. Arroyo* [1], *Mario Blázquez-Sánchez* [4,5], *David Rodríguez-Temporal* [4,5], *Belén Rodríguez-Sánchez* [4,5], *Luis Mancera* [1], *Coral del Val* [2,3*]

Corresponding autor email:delval@ugr.es

[1] Clover Bioanalytical Software S.L, 18016, Granada, Spain

[2] Department of Computer Science and Artificial Intelligence, Andalusian Research Institute in Data Science and Computational Intelligence (DaSCI), University of Granada, 18071 Granada, Spain.

[3] Instituto de Investigación Biosanitaria ibs.GRANADA, Complejo Hospitales Universitarios de Granada, Universidad de Granada, 18012 Granada, Spain.

[4] Clinical Microbiology and Infectious Diseases Department, Hospital General Universitario Gregorio Marañón, 28007, Madrid, Spain

[5] Institute of Health Research Gregorio Marañón, 28007, Madrid, Spain

TABLE OF CONTENTS

Figures:

Figure S1. Principal Component Analysis (PCA) of training data after ‘mean-based’ batch effect correction, highlighting two principal components

Figure S2. MALDI-TOF MS spectra after ‘mean’ batch effect correction. Representative spectra showing improved baseline and intensity uniformity compared with uncorrected data, demonstrating that the ‘mean’ ComBat approach partially mitigates inter-batch variability.

Figure S3. Common spectral peaks among *M. abscessus* subspecies after ‘var+mean’ correction with ClusterCentroids resampling and SVM.

Figure S4. Common spectral peaks among *M. abscessus* subspecies after ‘var.+mean’ correction with RandomOverSampler resampling and RF.

Figure S5. Comparison of discriminative peaks for *M. abscessus* subspecies between SVM (ClusterCentroids) and RF (RandomOverSampler) models after ‘var.+mean’ correction.

Figure S6. Comparison of discriminative peaks for *M. bolletii* subspecies between SVM (ClusterCentroids) and RF (RandomOverSampler) models after ‘var.+mean’ correction.

Figure S7. Comparison of discriminative peaks for *M. massiliense* subspecies between SVM (ClusterCentroids) and RF (RandomOverSampler) models after ‘var.+mean’ correction.

Figure S8. Principal component analysis (PCA) analysis of antibiotic resistance profiles in *M. abscessus* subspecies.

Figure S9. Average intensities per *M. abscessus* subspecies with standard deviation for primary discriminative m/z regions.

Tables:

Table S1. Dataset summary for the 325 *Mycobacterium abscessus* samples included in this study, categorized by subspecies, source hospital and extraction dates. (xls)

Table S2. List of selected peaks by subset using Boruta. (xls)

Table S3. Tested and selected hyperparameter configurations for classification algorithms. (xls)

Table S4. Performance metrics (and average performance) of the classification models on the validation data using 'var+mean' batch effect correction, Boruta feature selection and different sampling techniques. (xls)

Table S5. Performance metrics (and average performance) of the classification models on the validation data using 'mean' batch effect correction, Boruta feature selection and different sampling techniques. (xls)

Table S6. Top features intersections between *Mycobacterium abscessus* subespecies with 'var.+mean' correction and the combination of ClusterCentroids resampling and SVM. (xls)

Table S7. Comparation of top peaks between the two best combination of resampling methonds and classification models. (xls)

Table S8. Real Split reproduction for 13 isolates with available antibiotic susceptibility. (xls)

Figure S1. Principal Component Analysis (PCA) of training data after 'mean-based' batch effect correction, highlighting two principal components. Different colors represent distinct batches, each originating from one or more hospitals. Compared to uncorrected data, the sample distribution is notably more uniform, indicating effective batch effect mitigation.

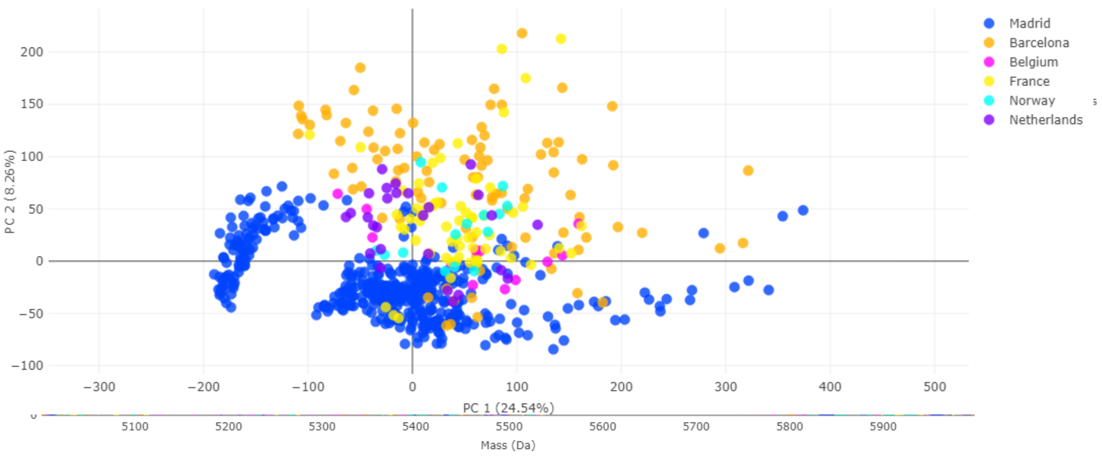

Figure S2. MALDI-TOF MS spectra after ‘mean’ batch effect correction. Representative spectra showing improved baseline and intensity uniformity compared with uncorrected data, demonstrating that the ‘mean’ ComBat approach partially mitigates inter-batch variability. Each color-coded line represents a batch from the dataset (Madrid, Barcelona, Belgium, France, Norway, and the Netherlands). Compared to the uncorrected data, peak alignment is notably improved, though it remains less pronounced than the alignment achieved by the ‘var.+mean’ correction method. Overall peak intensities tend to decrease, except for those initially lower in intensity—such as the 5650 m/z peak in the Madrid batch—which become more prominent after correction.

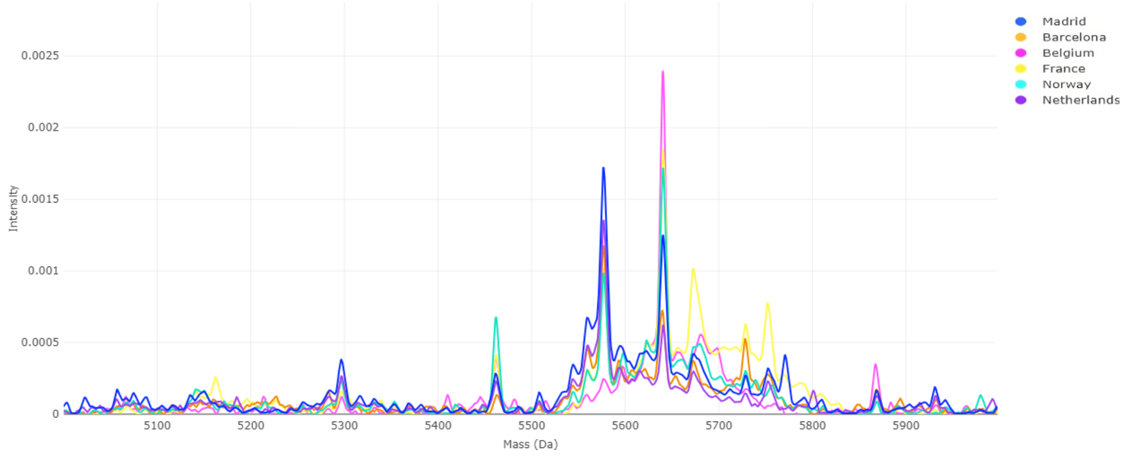

Figure S3. Common spectral peaks among *M. abscessus* subspecies after 'var.+mean' correction with ClusterCentroids resampling and SVM. Each coloured circle represents a subspecies (*M. abscessus, M. bolletii, M. massiliense*). The table shows the number of common peaks among them, obtained after applying the batch effect correction with the 'var+mean' method and the combination of the ClusterCentroids+SVM algorithms.

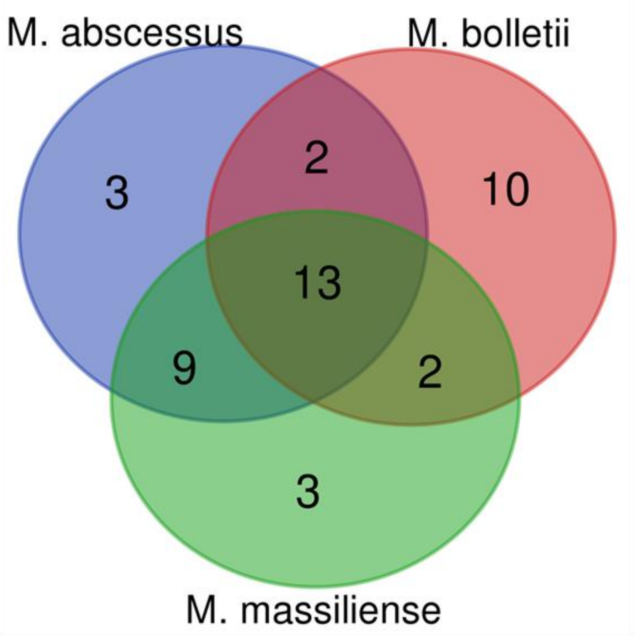

**Common Spectral Peaks Among *M. abscessus* Subspecies After 'var+mean' Batch Correction and ClusterCentroids Resampling and SVM**		
**Species**	**Total**	**Peaks**
M. abscessus and *M. bolletii*	2	2673.78, 3490.84
M. abscessus and *M. massiliense*	9	2671.25, 3108.79, 3106.18, 3106.70, 3107.74, 3119.22, 3107.22, 3105.66, 3108.26
M. bolletii and *M. massiliense*	2	2081.06, 2080.55
M. abscessus, *M. bolletii* and *M. massiliense*	13	3120.27, 3123.39, 2671.75, 3122.87, 3120.79, 3121.31, 2590.45, 3122.35, 2673.27, 2672.76, 3119.74, 3121.83, 2672.26

Figure S4. Common spectral peaks among *M. abscessus* subspecies after 'var.+mean' correction with RandomOverSampler resampling and RF. Each coloured circle represents a subspecies (*M. abscessus, M. bolletii, M. massiliense*). The table shows the number of common peaks among them, obtained after applying the batch effect correction with the 'var.+mean' method and the combination of the RandomOverSampler +RF.

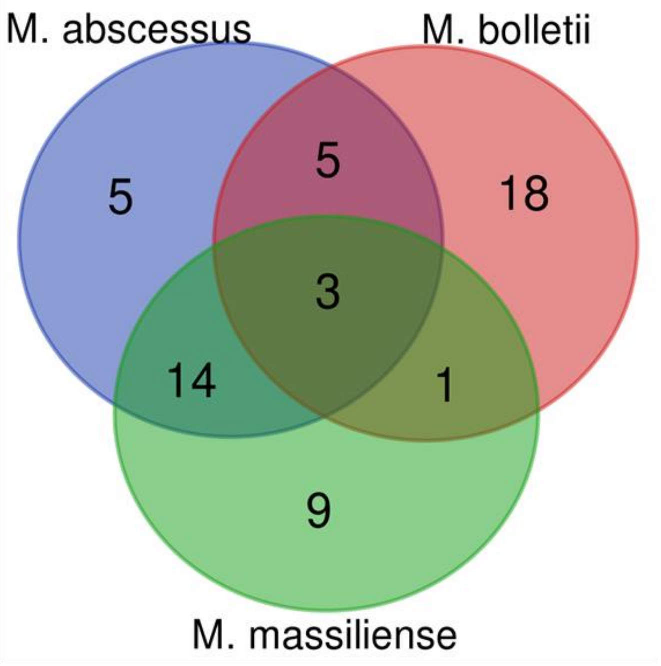

Common Spectral Peaks Among *M. abscessus* Subspecies After 'var+mean' Batch Correction and ROS Resampling and RF		
**Species**	**Total**	**Peaks**
M. abscessus, M. bolleti *and* *M. massiliense*	3	6962.62, 6964.18, 6962.11
M. abscessus and *M. bolletii*	5	5480.28, 5480.78, 3021.51, 6959.50, 6965.74
M. abscessus and *M. massiliense*	14	3123.91, 2081.58, 3120.27, 6960.54, 3123.39, 6960.01, 3119.22, 3122.87, 3120.79, 3121.31, 6963.67, 3119.74, 3121.83, 6961.57
M. bolletii and *M. massiliense*	1	3461.63

Figure S5. Comparison of discriminative peaks for *M. abscessus* subspecies between SVM (ClusterCentroids) and RF (RandomOverSampler) models after 'var.+mean' correction. Each coloured circle represents one of the two methodologies. The table shows the number of common peaks between the two methodologies for the same subspecies, obtained after applying the correction for batch effect with the 'var.+mean' method.

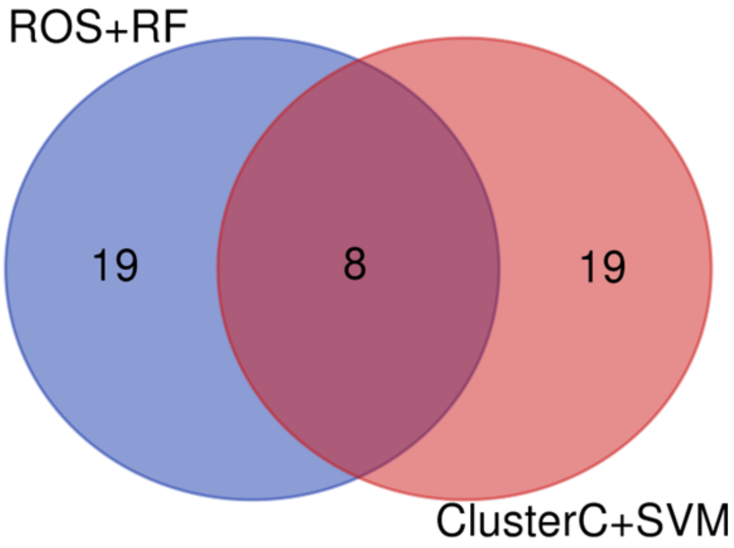

Common Spectral Peaks Among ROS Resampling with RF and ClusterCentroids Resampling with SVM After 'var+mean' Batch Correction for *M. abscessus* subspecie		
**Species**	**Total**	**Peaks**
M. abscessus ROS+RF and *M. abscessus* ClusterCentroids (ClusterC)+SVM	8	3123.91, 3120.27, 3123.39, 3122.87, 3120.79, 3121.31, 3119.74, 3121.83

Figure S6. Comparison of discriminative peaks for *M. bolletii* subspecies between SVM (ClusterCentroids) and RF (RandomOverSampler) models after 'var.+mean' correction. Each coloured circle represents one of the two methodologies. The table shows the number of common peaks between the two methodologies for the same subspecies, obtained after applying the correction for batch effect with the 'var.+mean' method.

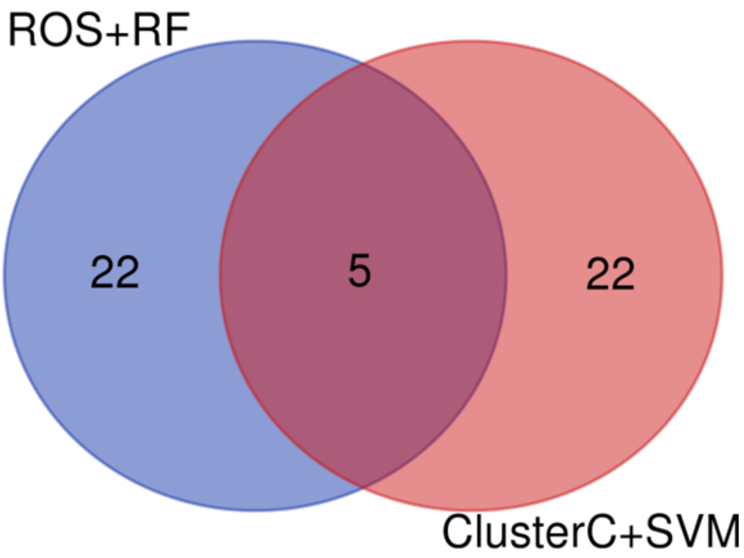

Common Spectral Peaks Among ROS Resampling with RF and ClusterCentroids Resampling with SVM After 'var+mean' Batch Correction for *M. bolletii* subspecie		
**Species**	**Total**	**Peaks**
M. bolletii ROS+RF and *M. bolletii* ClusterCentroids (ClusterC)+SVM	5	3024.07, 3462.13, 3022.53, 3021.51, 3023.56

Figure S7. Comparison of discriminative peaks for *M. massiliense* subspecies between SVM (ClusterCentroids) and RF (RandomOverSampler) models after 'var.+mean' correction. Each coloured circle represents one of the two methodologies. The table shows the number of common peaks between the two methodologies for the same subspecies, obtained after applying the correction for batch effect with the 'var.+mean' method.

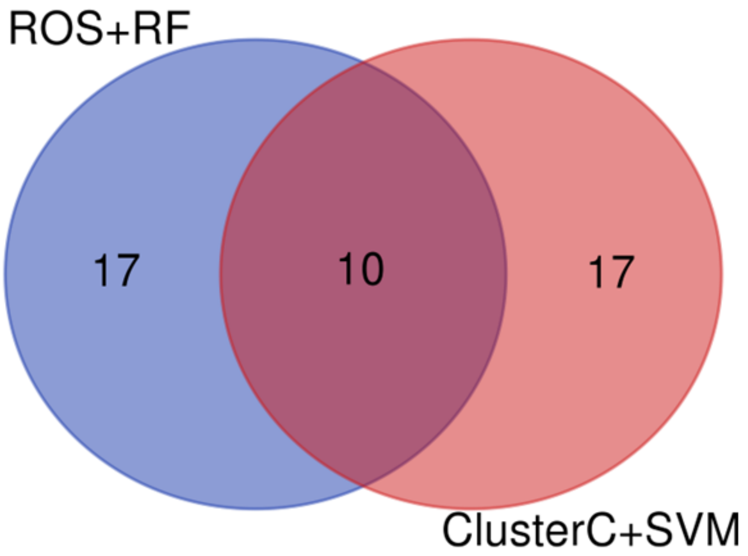

Common Spectral Peaks Among ROS Resampling with RFand ClusterCentroids Resampling with SVM After 'var+mean' Batch Correction for *M. massiliense* subspecie		
**Species**	**Total**	**Peaks**
M. massiliense ROS+RF and *M. massiliense* ClusterCentroids (ClusterC)+SVM	10	3123.91, 3120.27, 3123.39, 3122.87, 3120.79, 3121.31, 3122.35, 2080.55, 3121.83, 3119.74

Figure S8. Principal component analysis (PCA) analysis of antibiotic resistance profiles in *M. abscessus* subspecies. PCA of MALDI-TOF MS spectra from all 325 isolates, with the 13 isolates (from Gregorio Marañón General University Hospital) having antibiotic susceptibility testing (AST) data highlighted by resistance profile to amikacin (AMK) and clarithromycin (CLA). Black points represent the 312 isolates without AST data. (a) *M. massiliense* detail: Isolates with different resistance profiles (AMK_R_CLA_S, AMK_S_CLA_R, AMK_S_CLA_S) are interspersed within a single subspecies cluster. (b) *M. bolletii* detail: Resistant and susceptible isolates co-localize without forming resistance-based subgroups. (c) *M. abscessus* subsp. *abscessus* detail: Overlapping distributions of resistant and susceptible isolates confirm that subspecies-specific proteomic signatures dominate over resistance-related differences. Minor dispersion reflects natural biological and technical variability rather than consistent resistance-associated features.

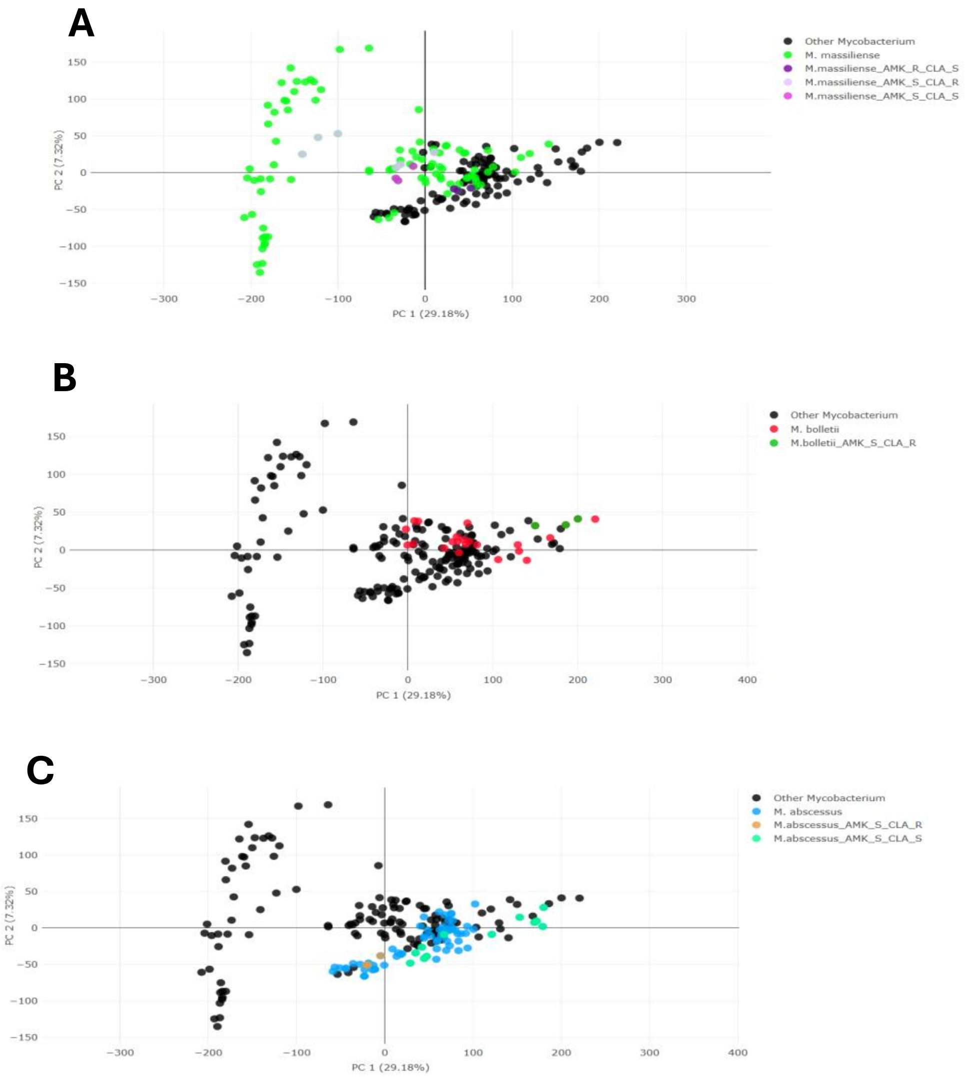

Figure S9. Average intensities per *M. abscessus* subspecies with standard deviation for primary discriminative m/z regions. (A) Average intensities per *M. abscessus* subspecies for mass ~2672. (B) Average intensities per *M. abscessus* subspecies for mass ~3105. (C) Average intensities per *M. abscessus* subspecies for mass ~3120. Different colors represent three subspecies of *M. abscessus* (*M. abscessus*, *M. bolletii* and *M. massiliense*).

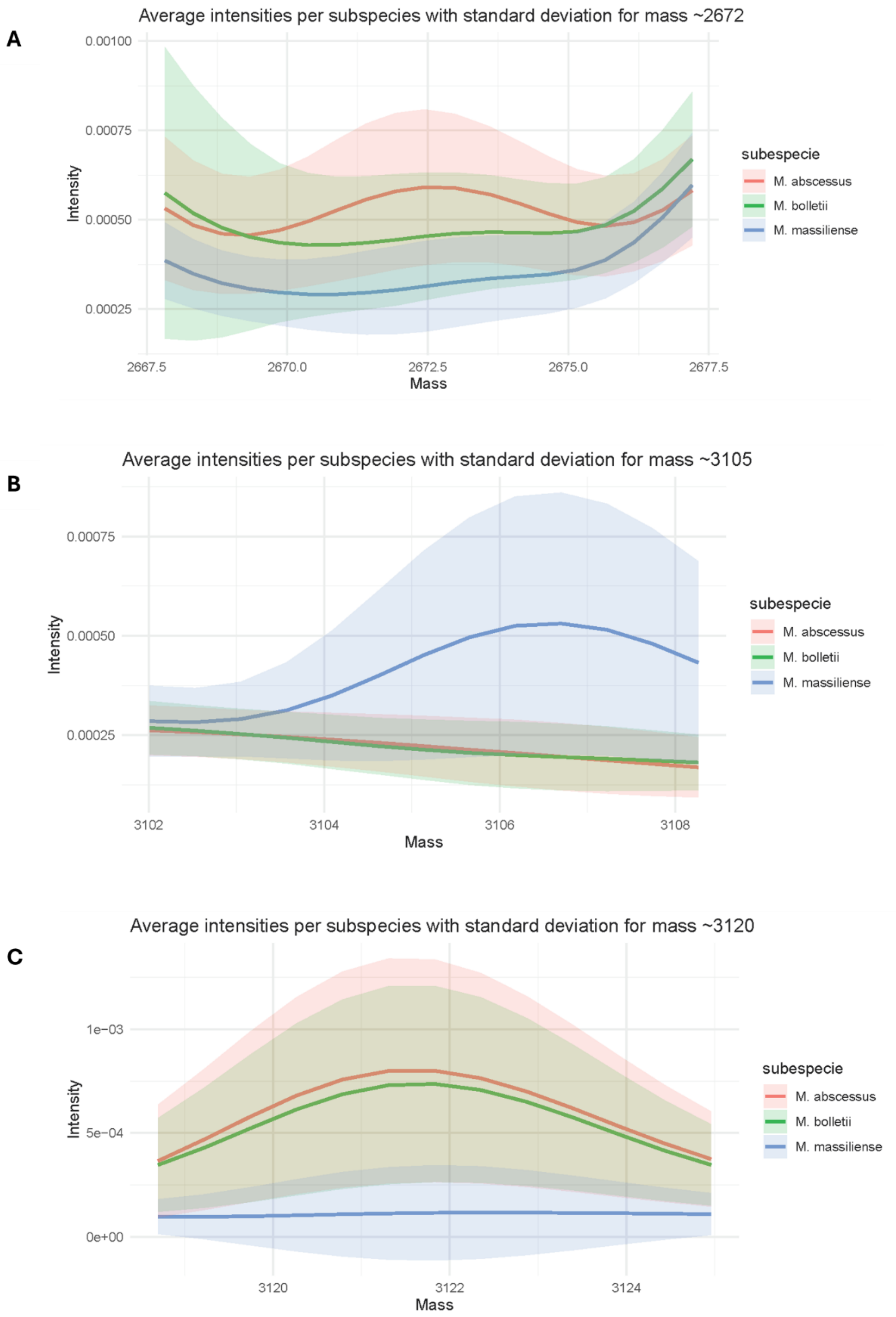

Supplement: Supplementary file 1 [file pr5c00534_si_001.pdf]
